# The impact of digital health on loneliness and social isolation for older adults: A scoping review protocol

**DOI:** 10.1371/journal.pone.0355296

**Published:** 2026-08-14

**Authors:** Chandler Tyrrell, Sinéad Keogh, Brídín Carroll

**Affiliations:** 1 Department of Social Science, Atlantic Technological University, Sligo, Ireland; 2 Department of Business, Atlantic Technological University, Galway, Ireland; 3 Irish Centre for Social Gerontology, University of Galway, Galway, Ireland; University of Porto, Faculty of Medicine, PORTUGAL

## Abstract

As populations age and healthcare systems increasingly adopt digital health technologies and alternative models of service delivery, understanding their broader social impact is becoming increasingly important. Digital health technologies are transforming how older adults access and manage healthcare services and may help alleviate pressure on healthcare systems. However, there is limited research examining whether digital health technologies mitigate or exacerbate experiences of loneliness and social isolation among older adults. Loneliness refers to the subjective feeling of inadequate social connection, while social isolation denotes the objective absence of social relationships. Both are associated with poorer physical and mental health, increased healthcare utilisation, and become more prevalent in later life. This protocol outlines a scoping review that will map the existing literature on the relationship between digital health technologies and loneliness and social isolation among older adults. The review will identify the types of digital health technologies that have been studied, examine how loneliness and social isolation have been defined and measured, and highlight gaps in the current evidence base to inform future research, policy, and practice. To our knowledge, this will be the first scoping review to comprehensively examine the intersection of digital health, loneliness, and social isolation in older adults. The protocol has been preregistered on the Open Science Framework (OSF Registries, available at: https://doi.org/10.17605/OSF.IO/92FPE).

## Introduction

The global population of adults aged 65 years and older is projected to double to approximately 1.5 billion by 2050, presenting significant challenges for health systems worldwide [1]. Rising life expectancy, driven by advances in healthcare and medical technology, represents a major achievement in modern medicine [2]. However, increased longevity is accompanied by a growing prevalence of age-related chronic conditions, including cardiovascular disease, stroke, diabetes, and neurodegenerative disorders, contributing to increasing multimorbidity and complexity of care [3–5]. These demographic and epidemiological trends underscore the need for adapted care models and innovative service delivery approaches.

In this context, digital health innovations, including telemedicine and mobile health (mHealth), electronic health records (EHRs), wearable devices and artificial intelligence are increasingly promoted as mechanisms to enhance healthcare access, support self-management, and alleviate pressures on strained health systems [6–8]. These technologies may influence experiences of loneliness and social isolation by changing how older adults access healthcare, communicate with healthcare professionals, and engage with health services and social support, although the nature and extent of these impacts remain unclear.

Digital health refers to the use of digital tools and services to support health delivery and promote well-being at individual and population levels [9–10]. For older adults, these technologies may facilitate ageing in place, support continuity of care, and enable sustained engagement with health services and communities [6,7,10,11]. However, older adults also face well-documented barriers to accessing and using digital technologies, including limited digital literacy, affordability constraints, and usability challenges, which may exacerbate existing health and social inequalities [12–14].

Loneliness and social isolation are increasingly recognised as urgent public health challenges, receiving growing attention from researchers, policy makers, and international organisations [8,15]. Loneliness is a subjective, distressing experience arising from a perceived discrepancy between an individual’s desired and actual social relationships [16–17]. In contrast, social isolation is an objective, quantifiable state characterised by limited social contact, a reduced social network, and infrequent social interaction [18–19]. Both tend to become more prevalent with age, and the COVID-19 pandemic further highlighted the vulnerability of older adults by restricting social contact [20–21]. Existing literature suggests loneliness and social isolation are associated with poorer physical and mental health outcomes, including increased risk of mortality [19,22]. In addition, severe loneliness has been associated with increased primary care visits, emergency department use, and polypharmacy, further underscoring the clinical relevance of loneliness and the potential importance of digital health interventions in addressing this issue [23].

Despite the rapid expansion of digital health solutions, their influence on loneliness and social isolation among older adults remains unclear. Some digital tools may enhance social connection and access to social support, whereas others may inadvertently reduce face to face interactions or deepen exclusion among those who are unable to engage with digital technologies. To date, the literature examining the relationship between digital health, loneliness and social isolation remains fragmented.

This protocol outlines a scoping review that aims to map the available literature on the impact of digital health technologies on older adults’ experiences of loneliness and social isolation. A scoping review methodology is well suited to this topic given the wide spectrum of digital health tools and implementation contexts, the anticipated heterogeneity in study designs and outcome measures, and the subjective nature of loneliness alongside the more objective measurement of social isolation. It is particularly appropriate for examining how loneliness and social isolation are conceptualised and measured within digital health research, and for identifying patterns and gaps across this emerging field. To assess the novelty of the review, an initial scoping search of key bibliographic databases and review sources was undertaken using combinations of terms related to digital health, older adults, loneliness, social isolation and social outcomes. Search terms were iteratively refined to capture relevant synonyms and related concepts, and reference lists of key papers were screened. This preliminary search did not identify any prior review that directly addressed this specific intersection.

## Materials and methods

This scoping review was developed in accordance with the methodological guidance and protocol template outlined by Lely et al. [24]. It will follow the framework originally proposed by Arksey and O’Malley [25], with methodological refinement from Levac et al. [26] and will align with the recommendations of the Joanna Briggs Institute (JBI) Manual for Evidence Synthesis [27]. Reporting of the completed review will be guided by the Preferred Reporting Items for Systematic Reviews and Meta-Analyses extension for Scoping Reviews (PRISMA-ScR) checklist [25]. The PCC (Population, Concept, Context) framework, as recommended by the JBI Manual for Evidence Synthesis [27], guided the development of the inclusion and exclusion criteria for this protocol.

The decision to undertake a scoping review rather than a systematic review was informed by Munn et al. [28], who note that systematic reviews are typically designed to address focused questions regarding the effectiveness of specific interventions or practices. In contrast, scoping reviews are appropriate for mapping the extent, range, and nature of available evidence, clarifying key concepts, and identifying knowledge gaps [25–27]. Given the anticipated heterogeneity in digital health interventions, study designs, and outcome measures related to loneliness and social isolation, a scoping review was considered the most appropriate methodology to provide a structured overview of the existing evidence and identify areas requiring further investigation.

### Review questions

1. What evidence exists regarding the impact of digital health technologies on loneliness and social isolation among older adults?
2. How are digital health technologies, loneliness and social isolation defined and conceptualised within the literature?
3. What populations or subpopulations of older adults are represented in the literature on digital health technologies and loneliness or social isolation?

### Participants

The review will consider studies involving older adults aged 65 years and over. Although alternative age thresholds are used in some literature, including 60 years in World Health Organisation guidance and other context specific definitions, 65 years was selected to align with the Medical Subject Headings (MeSH) indexing and to promote consistency and comparability with the broader literature. Eligible studies will include those in which loneliness and/or social isolation are examined as outcomes, experiences, or constructs of interest. Studies involving older adults with a range of health statuses, including physical, cognitive, and chronic health conditions, will be eligible. Mixed-age studies will also be included where data relating specifically to participants aged 65 years and over are reported separately. Where such data are not reported, two reviewers will independently assess whether sufficient age-specific detail is available for inclusion, with disagreements resolved through discussion and, where necessary, consultation with a third reviewer.

### Concept

The concept addressed in this scoping review is the impact of digital health on older adults’ experiences of loneliness and social isolation. As terminology varies across the literature, the search strategy will incorporate core terms including digital health, eHealth, mHealth, telehealth, telemedicine, and other digitally enabled health interventions; the full search strategy and complete list of terms are provided in Supporting Information. The search strategy was peer reviewed in accordance with the PRESS guideline to support transparency and methodological rigour. Eligible studies will include those in which loneliness and/or social isolation are examined as outcomes, experiences, or primary constructs of interest. These constructs may be assessed using validated instruments (e.g., the UCLA Loneliness Scale), clearly described indicators, proxy measures, or qualitative operationalisations, provided that they are explicitly linked to loneliness and/or social isolation in the study and are described in sufficient detail to permit transparent charting and interpretation.

### Context

No restrictions will be placed on geographical location, healthcare setting, or social context. Studies conducted in any setting, including community, primary care, hospital, long-term care, and home-based environments, will be eligible, provided they examine digital health in relation to loneliness and/or social isolation among older adults. Only studies published in English will be included, which may limit the representation of evidence from non-English-speaking settings where digital health adoption and ageing demographics are particularly salient. The review will initially consider literature published between 1 January 2015 and 1 November 2025, a period selected to reflect the rapid development and maturation of digital health technologies. The search will be rerun prior to final analysis to capture more recent studies, and the final search end date will be reported in the completed review, with any necessary updates made to the registration record.

### Types of sources

This scoping review will consider quantitative, qualitative, or mixed method studies that examine digital health in relation to loneliness and/or social isolation amongst older adults. Eligible study designs include randomised controlled trials, quasi-experimental studies, cohort studies, case-control studies, cross-sectional studies, and qualitative research. Several categories of source were considered and will be excluded from this review. First, case reports and single-participant studies will be excluded, as their findings are not generalisable beyond the individual case and do not provide the methodological detail required to meaningfully chart intervention characteristics and outcomes across the broader evidence base. Second, review articles, including systematic reviews, scoping reviews, and narrative or literature reviews, will be excluded to ensure that the review focuses on primary empirical evidence and avoids duplication of findings synthesised elsewhere, consistent with recommended scoping review methodology. To confirm the appropriateness of this approach, exploratory searches of related reviews were undertaken to assess whether this specific research question had already been addressed and to ensure that the review scope was distinct from, rather than duplicative of, prior synthesis work. Reference lists of relevant reviews identified during screening will also be hand-searched to identify additional eligible primary studies not captured through the database search strategy. Finally, conference abstracts, editorials, and opinion pieces will be excluded, as they do not report sufficient methodological detail or original empirical data to meet the data charting requirements of this review. Together, these exclusions ensure the review draws on primary empirical evidence appropriate for mapping the breadth and characteristics of the existing literature.

### Exclusion criteria

Studies not published in English will be excluded. The review will be restricted to literature published from 1 January 2015, up to 1 November 2025. This time frame was selected to reflect the rapid expansion and maturation of digital health technologies in recent years.

### Search strategy

A comprehensive search strategy was developed to identify key words contained in the titles and abstracts of peer-reviewed literature relating to digital health, loneliness, social isolation, and older adults. The primary reviewer developed the initial search strategy, which was subsequently refined in consultation with a senior social science research librarian at Atlantic Technological University (ATU). The search strategy was developed in accordance with the Peer Review of Electronic Search Strategies (PRESS) guideline, and the full search strategies are provided in Supporting Information. The electronic databases PubMed, MEDLINE, IEEE Xplore, Web of Science, and Scopus were selected to ensure comprehensive coverage of health, social science, and technology-related literature relevant to digital health and ageing. Grey literature sources, including government reports, World Health Organisation publications, and clinical trial registries, will not be searched, as the review seeks to map peer-reviewed empirical evidence while maintaining a focused and reproducible search strategy. In line with scoping review methodology, the search strategy prioritised sensitivity over specificity, using broad controlled vocabulary and free-text terms to maximise retrieval across disciplines while minimising the risk of missing potentially relevant studies. The search was executed on 1 November 2025 and identified 25,724 records prior to duplicate removal. At the time of protocol submission, screening and data extraction have not yet been completed, and no results have been generated. To maintain the currency of the review, the search will be re-executed by the primary reviewer immediately prior to final data analysis; any additional records identified after the original 1 November 2025 cut-off will be screened using the same eligibility criteria and, where eligible, incorporated into the review. The revised search date will be reported in the completed review, and the OSF registration will be updated accordingly to ensure transparency and consistency between the protocol and the final review.

### Study/Source of evidence selection

All identified citations were uploaded into the Covidence software platform [29], and duplicates will be removed. Titles and abstracts will be screened by one reviewer against the predefined inclusion criteria. Full-text articles of potentially eligible studies will then be retrieved and assessed for inclusion in the review. To minimise potential selection bias and assess consistency in the application of the eligibility criteria, a second reviewer will independently screen a random subset (20%) of records at both the title/abstract and full-text stages. This threshold was considered sufficient to provide a meaningful check on the consistency of screening decisions while remaining feasible and proportionate to the large volume of records retrieved. Inter-rater reliability for the independently screened subset will be calculated and reported using Cohen’s kappa [30], as this statistic provides a standard measure of agreement beyond chance and is widely used in evidence synthesis methodology. Any disagreements will be resolved through discussion, with consultation from a third reviewer where necessary. The study selection process will be comprehensively documented in the final scoping review manuscript and presented using a PRISMA flow diagram [27] to ensure transparency.

### Data extraction

Data will be charted by the primary reviewer using a data charting form developed in accordance with the recommendations of the JBI Manual for Evidence Synthesis [27]. Prior to full data charting, the charting form will be pilot tested on five included studies to ensure clarity, consistency and a shared understanding of the charting categories. These five studies will remain in the review and will be included in the final data charting following any necessary refinements to the charting form. The charting form will be used to record key characteristics of the included studies, including author(s) and year of publication; geographical location; research aims and objectives; study design; summary of findings; and definitions and measures related to digital health, loneliness, and social isolation. To enhance methodological rigour, a second reviewer will independently verify the accuracy and completeness of the charted data for a random subset of 20% of included studies. Agreement for the verified subset will be assessed using Cohen’s kappa [30], with a value of 0.60 or higher considered acceptable agreement. Where agreement falls below this threshold, discrepancies will be reviewed, any necessary refinements will be made to the charting form, and the affected studies will be re-examined. Any unresolved discrepancies will be settled through discussion and, where necessary, adjudication by a third reviewer. Consistent with the iterative nature of scoping reviews, the data charting form may be refined during the review process, with any modifications documented and justified in the final manuscript.

### Data analysis

NVivo (version 14) will be used to support coding and analyse of data from the studies included in this scoping review. Descriptive summary statistics will also be used to map the characteristics of included studies. Frequency counts will be generated for key variables such as year of publication, geographical location, study design, type of digital health technology, and measurement instruments used. These findings will be presented in tabular and graphical formats where appropriate to provide a structured overview of the evidence base. Qualitative data relating to digital health, loneliness, and social isolation will be analysed using a hybrid deductive-inductive thematic approach. An initial coding framework will be derived from the review objectives and key a priori concepts, including definition, conceptualisation, and measurement, and additional inductive coding will be applied to capture emergent categories not fully represented in the framework. This approach aligns with established thematic analysis methods that integrate theory-driven and data-driven coding in qualitative synthesis [31]. During data charting and analysis, the concepts of digital health, loneliness, and social isolation will be examined across three analytically distinct but related domains: definition, conceptualisation, and measurement. For the purposes of this review, definition will refer to the explicit meaning assigned to digital health, loneliness, or social isolation within a study, including author-provided definitions or cited conceptual sources. Conceptualisation will refer to the broader theoretical or interpretive framework through which these constructs are understood or positioned, such as digital inclusion and exclusion frameworks, technology acceptance models, ageing in place perspectives, social connectedness theories, or life-course approaches. Measurement will refer to how loneliness and social isolation are operationalised and assessed, including the use of validated scales, bespoke survey items, proxy indicators, or qualitative operationalisations. To support consistency in coding, these distinctions will be defined in the data charting form and applied throughout the analysis. The qualitative synthesis will proceed iteratively, with categories refined as coding progresses and as new insights emerge from the included studies. These domains will be charted separately and synthesised narratively to identify patterns, consistencies, and gaps in how digital health and social outcomes are defined, conceptualised, and measured within the existing literature. Consistent with scoping review methodology, any refinements made to the analytical framework during the review process will be documented and transparently reported in the final manuscript.

## Discussion

The primary objective of this scoping review is to map and synthesise the available evidence on the impact of digital health on loneliness and social isolation for older adults. A secondary objective is to identify and examine how these experiences are conceptually defined and measured within existing literature. Identifying and documenting definitions and measurement approaches will support greater conceptual clarity, improve comparability across studies, and enhance the quality, and reproducibility of future research. This process will facilitate the identification of knowledge gaps. Such work has the potential to foreground theory-informed policy development and support the design and more methodologically robust digital health interventions. Planned dissemination will include preparation of a policy briefing targeted at decision makers and stakeholder organisations working in healthy ageing, digital health, and social care, including national health departments, public health agencies, and relevant international bodies such as the World Health Organization and the European Commission, as appropriate. Findings will also be disseminated through submission of a full journal article to a peer-reviewed outlet. To support broader knowledge translation, a plain language summary will also be developed to communicate the review findings in an accessible format for older adult communities, advocacy organisations, and other non-academic audiences. This scoping review will be subject to several anticipated limitations. First, inclusion will be restricted to studies published in English language, which may result in the exclusion of relevant non-English language studies. Second, the review will be limited to peer-reviewed literature and will exclude grey literature, which may result in the omission of relevant reports, policy documents, theses, conference proceedings, or other unpublished sources and may increase the risk of publication bias, as studies with neutral or negative findings are less likely to appear in the published literature. Third, given the dynamic and rapid pace of innovation in digital health, limiting the search to publications between 1 January 2015–1 November 2025, may mean that the most recent developments are not captured. Fourth, data extraction will be conducted by a single primary reviewer, with a sample of 20% of included studies independently verified by a second reviewer; although this approach is pragmatic and used in some constrained review processes, it may increase the possibility of extraction error or inconsistency compared with full dual independent extraction. However, as the focus of the review is to map the breadth and nature of literature on the topic, rather than to synthesise the best available evidence, these limitations are considered acceptable within the context of the review objectives. Despite these limitations, the completed scoping review is expected to provide a comprehensive overview of the current evidence base, highlight methodological and conceptual gaps, and inform future research, policy, and practice related to the design and evaluation of digital health interventions aimed at supporting social wellbeing in later life.

## Supporting information

S1 Fig PRISMA-P (Preferred Reporting Items for Systematic review and Meta-Analysis Protocols). (DOCX)

S2 Fig Search strategy; PubMed. (DOCX)

S3 Fig Data extraction instrument. (DOCX)
