# Supplementary material for: The impact of digital health on loneliness and social isolation for older adults: A scoping review protocol
Source: PLoS One. 2026 Aug 14;21(8):e0355296. doi: 10.1371/journal.pone.0355296 (PMC13475860; doi:10.1371/journal.pone.0355296)
Supplement: Supplementary file 2 [file pone.0355296.s002.docx]

### Appendix II: Search strategy; PubMed

(("aged"[Mesh] OR "aging"[Mesh] OR "aged"[tiab] OR "aging"[tiab] OR "ageing"[tiab] OR "older adult*"[tiab] OR "older people"[tiab] OR "elderly"[tiab] OR "senior*"[tiab] OR "aging population"[tiab] OR "older age"[tiab] OR "later life"[tiab]) AND ("loneliness"[MeSH] OR "loneliness"[tiab] OR "lonely"[tiab] OR "social isolation"[MeSH] OR "social isolation"[tiab] OR "socially isolated" OR "social exclusion"[tiab] OR "socially excluded"[tiab] OR "social connectedness"[tiab] OR "social engagement"[tiab])) AND ("digital health"[MeSH] OR telemedicine[mesh] OR "digital health"[tiab] OR "telemedicine"[tiab] OR "telehealth"[tiab] OR "digital health technolog*"[tiab] OR "digital health services"[tiab] OR "mobile health"[tiab] OR "digital intervention*"[tiab] OR "digital technolog*"[tiab] OR "health information technolog*"[tiab] OR "ehealth"[tiab] OR "mhealth"[tiab] OR "health technology"[tiab])

| **#** | **Concept** | **Results** |
| --- | --- | --- |
| 1. Older People | "aged" OR “aging” OR “ageing” OR "older adult*" OR "older people" OR “elderly” OR “senior*” OR “aging population” OR “ageing population” OR “older age” OR “seniors” OR “later life” | *1,584,850* |
| 2. Loneliness or Social Isolation | "loneliness" OR "lonely" OR "social isolation" OR "socially isolated" OR "social exclusion" OR "socially excluded" OR “social connectedness” OR “social engagement” | *40,785* |
| 3. Digital Health | “digital health” OR “telemedicine” OR “digital health technolog*” OR “digital health services” OR “telehealth” OR “mobile health” OR “digital interventions” OR “digital technolog*” OR “health information technolog*” OR “ehealth” OR “mhealth” OR “health technology” | *104,366* |
| 4. | #1 AND #2 AND #3 | *371* |
