# Supplementary material for: The impact of digital health on loneliness and social isolation for older adults: A scoping review protocol
Source: PLoS One. 2026 Aug 14;21(8):e0355296. doi: 10.1371/journal.pone.0355296 (PMC13475860; doi:10.1371/journal.pone.0355296)
Supplement: Supplementary file 3 [file pone.0355296.s003.docx]

### Appendix III: Data extraction instrument

Data extraction elements

**Evidence Source Details and Characteristics**

Citation details

Title

Year

Authors

Country

Context

Participant details

Age

Disease related information

**Details extracted from the source of evidence**

Research aims and objectives

Study design classification

(Quantitative, Qualitative, Mixed Methods)

Summary of findings

1. Identify definitions and measures of Loneliness, Social Isolation and Digital Health.
2. Identify gaps for future research.
